# Conditions for the Evolution of Gene Clusters in Bacterial Genomes

**DOI:** 10.1371/journal.pcbi.1000672

**Published:** 2010-02-12

**Authors:** Sara Ballouz, Andrew R. Francis, Ruiting Lan, Mark M. Tanaka

**Affiliations:** 1 School of Biotechnology and Biomolecular Sciences, University of New South Wales, Kensington, New South Wales, Australia; 2 School of Computing and Mathematics, University of Western Sydney, Parramatta, New South Wales, Australia; 3 Evolution and Ecology Research Centre, University of New South Wales, Kensington, New South Wales, Australia; University of California Davis, United States of America

## Abstract

Genes encoding proteins in a common pathway are often found near each other along bacterial chromosomes. Several explanations have been proposed to account for the evolution of these structures. For instance, natural selection may directly favour gene clusters through a variety of mechanisms, such as increased efficiency of coregulation. An alternative and controversial hypothesis is the selfish operon model, which asserts that clustered arrangements of genes are more easily transferred to other species, thus improving the prospects for survival of the cluster. According to another hypothesis (the persistence model), genes that are in close proximity are less likely to be disrupted by deletions. Here we develop computational models to study the conditions under which gene clusters can evolve and persist. First, we examine the selfish operon model by re-implementing the simulation and running it under a wide range of conditions. Second, we introduce and study a Moran process in which there is natural selection for gene clustering and rearrangement occurs by genome inversion events. Finally, we develop and study a model that includes selection and inversion, which tracks the occurrence and fixation of rearrangements. Surprisingly, gene clusters fail to evolve under a wide range of conditions. Factors that promote the evolution of gene clusters include a low number of genes in the pathway, a high population size, and in the case of the selfish operon model, a high horizontal transfer rate. The computational analysis here has shown that the evolution of gene clusters can occur under both direct and indirect selection as long as certain conditions hold. Under these conditions the selfish operon model is still viable as an explanation for the evolution of gene clusters.

## Author Summary

Genes involved in a common pathway or function are frequently found near each other on bacterial chromosomes. A number of hypotheses have been previously presented to explain this observation. A particularly influential theory is the selfish operon model, which posits that horizontal transfer could promote gene clustering by favouring transfer of arrangements of genes that are close together. Subsequent theoretical development and analysis of genomic data have contributed to the debate about the plausibility of this model. Here, by re-examining the evolutionary dynamics of gene clusters, we provide and discuss conditions under which gene clusters can evolve. We find that first, some form of bias for clustering is required for clusters to evolve. This bias can be in the form of bias in horizontal transfer towards genes that are close together, or direct natural selection for gene proximity. Our computational work does not present a theoretical obstacle to the selfish operon model as a possible explanation for the evolution of gene clusters.

## Introduction

A conspicuous feature of bacterial genomes is the grouping of genes involved in a metabolic pathway into functional units on the chromosome. Early linkage studies of *Escherichia coli* and *Salmonella typhimurium* showed that genes in the biosynthetic pathways of tryptophan and histidine occur on a contiguous region of the genome [1],[2]. Furthermore, genes are often found in their biochemical reaction order [3]. Gene clustering has since become recognized as a widespread feature of bacterial genomes. Grouped genes are sometimes transcribed together as an operon, with shared promoter and operator sequences (for example the galactose operon *galETK* [4],[5]). Regulatory genes have also been found close to the genes they regulate. A classic example is the *lacI* repressor gene, which resides near but not within the *lacZYA* operon in *Escherichia coli*. The extent of gene clustering is variable – a given set of related genes may be clustered in one species but unclustered and/or reordered in another [6],[7]. Interestingly, most clusters do not contain much intergenic DNA, and in some cases genes even overlap [8],[9].

A number of explanations for clustering have been considered over the years. The most controversial and influential hypothesis has been the *selfish operon model*, which offers a mechanism for the evolution of clustering without needing to invoke the action of natural selection [10],[11]. In this model, gene clusters persist because the proximity of the genes in question facilitates their collective transfer between species. It applies to genes encoding accessory functions rather than essential genes.

Another model that does not require direct selection to explain clustering is the *persistence model* [12]. Unlike the selfish operon model, this model has been proposed to explain the clustering of essential genes – genes that are evolutionarily persistent. The hypothesis here is that by occupying less space, clustered genes are less likely to be disrupted by the deletion or insertion of DNA. In other words, an individual with clustered genes is more “resilient” to the lethal or deleterious effects of mutation. This hypothesis is similar to the idea that genes sharing regulatory sequences by residing in a single operon present a smaller target for deleterious mutation than scattered genes with individual control elements [13].

Hypotheses involving direct selection have also been examined. Here, clustering of genes confers a direct fitness advantage to the organism. For example, a scenario in which selection directly favouring the co-regulation of genes can lead to the evolution of operons has been outlined [14]. Apart from efficiently regulated transcription, a fitness advantage may arise through shorter diffusion times for proteins finding their targets when the genes encoding them are clustered. Thermodynamic models have been developed to apply this idea to enzymes and transcription factors [15],[16]. The efficiency gained from shorter diffusion times is assumed to translate into a reproductive fitness advantage [17]. Another mechanism conferring advantage to gene clustering is gene amplification [18]. In this model, gene dosage is rapidly and reversibly increased by tandem duplication of the genes in question. The closer the genes are, the greater the probability of coamplification. The increased dosage is assumed to contribute to elevated fitness. Other models for the evolution of gene clusters based on metabolic arguments have also been studied [19],[20].

Other hypotheses have been considered but rejected [10],[17, for example]. A hypothesis now called the *natal model* suggests that clusters arose by gene duplication and divergence such that the newly formed genes participate in a common pathway. However, the lack of sequence homology for most genes within clusters undermines this explanation [10]. Fisher's theory of the evolution of linkage and recombination has been suggested to apply to bacteria [1],[21]. Under this theory natural selection favours increased linkage among co-adapted genes – genes whose products work well together – because recombination (chromosomal crossover during meiosis) breaks up combinations of alleles with high fitness. However, it has been pointed out that this requires high recombination rates, which are typical for eukaryotes, to work [10]. Although recombination rates are found to be high in some species [22],[23], they are not high enough relative to the cellular generation rate to support an account of clustering based on Fisher's theory.

The debate on the origins and maintenance of gene clusters continues, with recent genomic studies casting doubt on the selfish operon hypothesis. First, the prediction that non-essential genes are clustered while essential genes are not has been tested and rejected [24]. Second, if horizontal gene transfer is an important source of gene clusters, then horizontally transferred sequences should be associated with operons. Genomic data, however, do not support such an association [14]. On the other hand, they do support the possibility that genes and their regulators may have evolved close proximity via horizontal transfer [25]. Third, the selfish operon model is unable to explain the observation that genes in clusters are sometimes arranged in the order of biochemical reactions. A resolution may involve multiple mechanisms, of which horizontal transfer of selfish operons is one [12].

Here, we re-examine the theoretical basis for explaining the origins and maintenance of gene clusters. By studying a number of distinct models, we provide and discuss conditions under which clustering can evolve.

## Model

We describe three kinds of models for gene clustering in this article. First, we revisit the selfish operon model [10]. We seek to explore the parameter space and understand in more detail when and why it produces gene clusters. Second, we propose a model based on the Moran process, which tracks individual bacterial cells and in which the total population size is constant. Third, we develop a further model that tracks the substitution of new arrangements, making the assumption that populations are monomorphic. By running computer simulations of these three systems we consider the factors that lead to the evolution of gene clusters.

The assumptions common to all models are as follows. Genomes are made up of circular chromosomes divided into 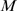 regions; we let 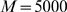 kilobases (kb). This genome size is constant over time. There are 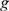 genes in the pathway of interest. Only a single gene can occupy any given position. The units of reproduction are either species or individual bacteria depending on the model. A genome can undergo rearrangement with probability 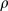 per step or generation. We explore two processes: first, translocation of a random gene to a random position and second, inversion by which two breakpoints are chosen randomly uniformly and the intervening segment inverted. If the resulting arrangement moves the terminus or origin more than 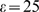 kb the new arrangement is regarded as lethal [26],[27]. Both translocation and inversion are used within the selfish operon framework of Lawrence and Roth 1996, while only inversion is considered for the Moran model and the rearrangement substitution model.

### Model of Lawrence and Roth 1996

In their influential paper, Lawrence and Roth describe a simulation model that produces gene clusters through a horizontal gene transfer process that is biased towards genes that are physically closer on a chromosome [10]. This is called the selfish operon model. In this model, species in which individuals carry all the genes needed for the function are called “positive” species. Each species is assumed to be monomorphically composed of genomes with a particular arrangement of genes on the chromosome, and fixation is assumed to occur instantaneously. That is, each species is associated with a single arrangement of genes. We are interested in the minimum arc distance along the chromosome that contains all genes, which is equivalently the genome length minus the longest interval between pairs of neighbouring genes. The simulation is initialised with 100 species, with each species given a random set of gene positions. Lawrence and Roth kept the number of species between 10 and 900 [10]. We have implemented this by switching off the horizontal transfer process when the number of species reaches 900 and re-instating it when the size goes under 900. We ran our simulations for 50,000 time steps.

Horizontal transfer leads to a species that lacks the function (a “negative” species) acquiring the function along with the arrangement of gene positions of the donor genome. The probability of horizontal transfer 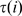 decreases with distance 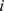. Although its form is not given in [10], we will assume it is exponential with a decay parameter 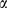. That is,
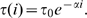
(1)The exponential distribution is a natural choice for the size distribution of transferred DNA among bacteria, and has been empirically tested for homologous recombination [28],[29]. Some support for a skewed distribution of gene transfer fragment lengths is found in Ochman and Jones 2000 [30].

At each time step, each species or individual can undergo loss of the function with probability 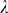. Following Lawrence and Roth, we set the loss probability 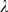 to 0.001 per genome per time step and the maximum probability of horizontal transfer 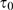, occurring when the genes are located in the same minute of the chromosome, to 0.01 per genome per time step [10]. We set 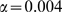 by default, under which a 50 kb fragment is 6 times more likely to transfer than one of 500 kb. Because the probability of rearrangement is likely to be very low in nature [31], we set 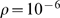 per genome per time step by default. Lawrence and Roth 1996 used a much higher value of 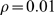 and we investigate the effect of lowering this parameter from this high value. We studied the effect of varying 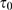 and 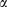 by varying parameters one at a time as well as using latin hypercube sampling [32],[33] to explore the parameter space. Under this methodology, each parameter is divided into equiprobable regions in the area of interest, and parameter sets are constructed by selecting values randomly from the resulting grid without replacement. A uniform distribution was used for each parameter.

The algorithm we used for the dynamic is as follows.

1. Initialise population as described above.
2. For each species 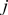,
  - With probability 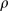, rearrange the genome by moving a gene to a random new position.
  - With probability 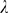, destroy gene function (the species is lost from the pool of positive species).
  - If the number of positive species is under 900:Horizontal transfer leads to recruitment of a species (from a limitless supply of negative species) with the same arrangement of genes as species 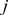, with probability 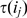, where 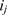 is the minimum arc distance between the genes in genome 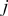.
3. Compute the average minimum arc distance between genes across species in the population of positive species.
4. Repeat steps 2, 3 until the end of the simulation.

One problem we have noticed with this model is that given a rearrangement event, the genes in question are always affected. A more natural assumption would be that the genes in question are affected with probability 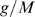, which is the proportion of the genome occupied by the 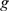 genes assuming that genes are 1 kilobase in length. Thus, we have also run the simulations using this corrected translocation process, replacing step 2(a) in the above algorithm with

2(a) With probability 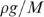 move a gene to a random new location.

This correction effectively lowers the rearrangement probability by a few orders of magnitude.

We have also implemented a version of the model in which rearrangement occurs by inversion instead of translocation. Here, we replace step 2(a) in the algorithm with

2(a) With probability 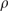 choose two random positions 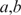 randomly uniformly between 1 and 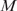. To implement breakpoints occurring between genes, subtract 0.5 from each of these values.

If (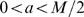 and 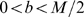) or (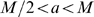 and 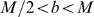) or (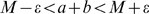) then the inversion is viable. (Recall 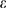 is the tolerance to imbalance between origin and terminus.) For each gene whose location 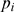 is between 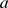 and 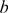, move it to its new location given by 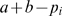.

### A Moran model of clustering

We construct a model in which the population evolves according to a Moran process [34],[35] combined with a process of genome inversion. Here, we track a population of bacterial cells. As with the selfish operon model, we consider a pathway involving 3 or more genes. A population is initialised with all bacteria carrying the same genome with genes placed randomly uniformly on the chromosome. The population size is 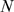. Let 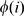 represent the relative fitness of cells with the genes at minimum arc distance 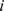. Genomes with the genes closer together have a reproductive or survival advantage over those with the genes further apart. We use the function 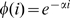 to describe this relationship. Because this relative fitness function is analogous to 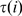, we use the same symbol (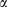) to describe the decay in fitness with respect to distance 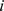. An alternative function 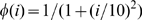 was also used to ascertain the effect of using a steep sigmoidal relationship. Selection for clustering here can be due to any of the mechanisms discussed in the Introduction.

The algorithm is as follows.

1. Initialise the population as described above.
2. Choose an individual at random. Choose two positions (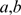) at random uniformly between 1 and 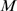. To implement breakpoints occurring between genes, subtract 0.5 from each of these values.
3. Inversion occurs with probability 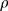.
  - If inversion occurs,
    - if (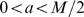 and 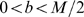) or (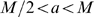 and 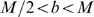) or (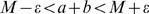) then the inversion is viable. For each gene whose location 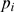 is between 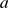 and 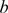, move it to its new location given by 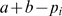.
    - Otherwise the inversion is lethal: replace the individual with a random individual from the population in proportion to 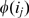 where 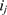 is the minimum arc distance between the genes in genome 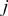.
  - Otherwise if inversion does not occur, there is random death and replacement. Replacement birth occurs by picking a random individual from the population in proportion to 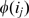.
4. Record the average minimum arc distance between genes across the population.
5. Repeat steps 2–4 until the end of the simulation.

Following the classical definition of the Moran process, a single generation is 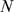 time steps.

This process is very slow with high population sizes, particularly when the rearrangement probability 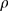 is low. The computational demands of running these simulations precluded the possibility of systematically analysing sensitivity to parameters. This motivated us to develop a further model, which tracks the mutation and fixation process without following details at the population level. This model is described in the next subsection.

### Rearrangement substitution model

Here, the population is monomorphic (except during periods of substitution of new arrangements) and so only a single genome arrangement is tracked. Again, the 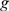 genes in the pathway in question can occupy 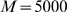 positions, 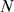 represents the population size and 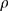 is the rearrangement probability. The assumption that the population is monomorphic implies that 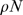 must not be too large. In each generation the probability of a rearrangement occurring in at least one individual is 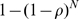 which can be approximated with 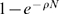 since 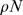 is small. The time until the next rearrangement event 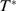 is distributed geometrically with parameter 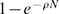. We use inversion rather than translocation as the source of rearrangements.

As above we specify selection through an exponential decay in fitness as a function of the minimum arc distance 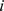, so that the *relative* fitness of a new genome with distance 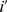 is 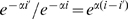, and the selective coefficient is 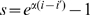. A new arrangement fixes in a population with probability
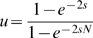
(2)and the time it takes to reach fixation is
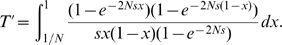
(3)These quantities have been derived from diffusion theory in population genetics (for details see [36]). We use 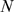 in place of 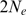 to apply the theory to haploids, where 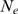 is the effective population size of a diploid population.

The algorithm for the rearrangement substitution model is therefore as follows.

1. Initialise by choosing a random arrangement of genes. Choose these positions without replacement.
   Set 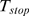, the number of generations to run the simulation. Set current time to 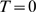.
2. Get random time 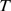 until next rearrangement event:
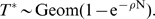

3. Inversion: choose two integers at random (uniformly between 1 and 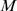 inclusive). Subtract 0.5 from each value. Label these points 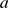 and 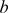.
  - If (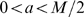 and 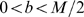) or (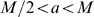 and 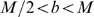) or (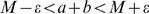) then the new arrangement is viable. Obtain the new arrangement as follows. Locate all genes between 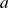 and 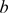. Call these positions 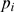. For each 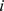, place the gene into the new location given by 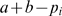. Go to Step 4.
  - Otherwise, the arrangement is not viable. Set 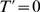 and go to Step 6.
4. Obtain the current minimum arc distance 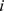 and the minimum arc distance 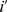 of the mutant arrangement. Compute the selection coefficient:
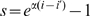
where 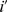 is the new distance.
5. Compute the fixation probability 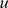 given by Equation (2). Fixation occurs if a random uniform U(0,1) is less than 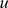.
   If fixation occurs, find the expected time until fixation 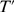 given by Equation (3). Set the current genome to the new arrangement.
  Otherwise there is no fixation and 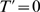.
6. Update elapsed time: 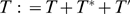

7. If elapsed time 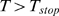, stop the process and record the minimum arc distance. Otherwise, return to Step 2.

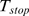
 was set at 50,000 generations. We investigated this model by varying one parameter at a time as well as using latin hypercube sampling to explore the parameter space.

## Results

### Lawrence and Roth model

**Figure 1:**
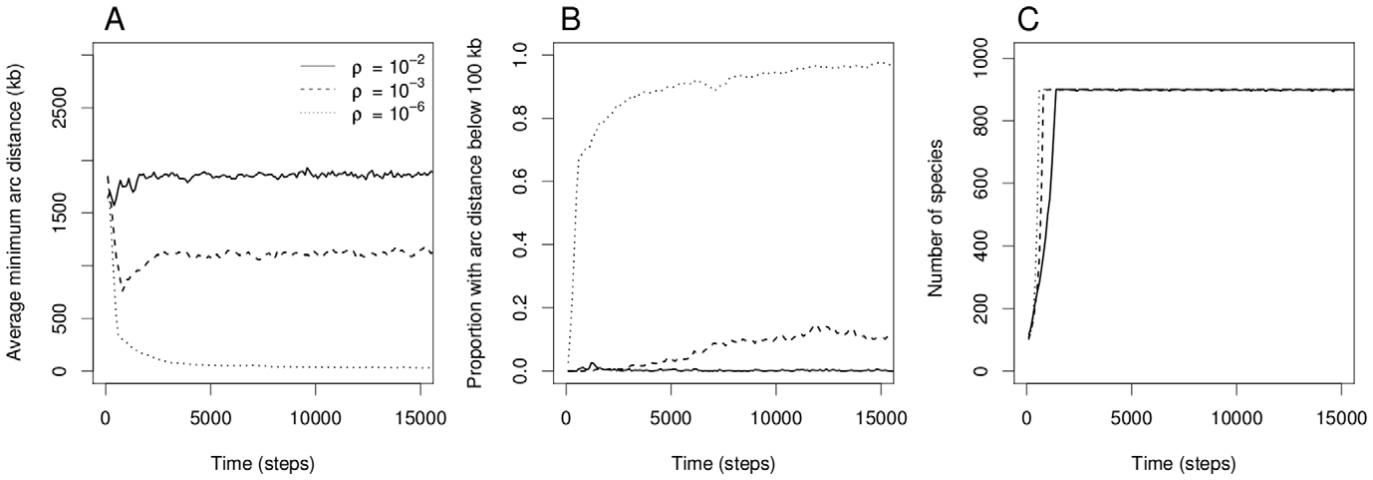
Gene clustering under the original selfish operon model. The plots show A) the average minimum arc distance between genes, B) the proportion of genes clustered under 3 minutes and C) the total population size over time, for three realisations of the process using three values of the rearrangement probability 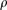, indicated in solid (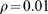), dashed (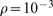) and dotted curves (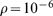). Unless indicated otherwise, there are three genes in the pathway and the parameter values are 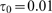, 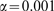 and 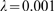. Only the first 15,000 steps of the simulations are shown here.

When three genes are placed randomly around a chromosome with a uniform distribution, the average minimum arc distance between them is around 1900 kb. When the rearrangement probability 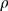 is 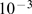 or 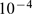, the selfish operon model [10] produces an initial wave of gene clustering down to around 600–800kb ((Figure 1A), also reflected in the rise of the proportion of genomes that are clustered under a threshold (Figure 1B). The maximum population size of 900 is reached quickly (Figure 1C) and the dynamics of clustering undergo a change as a new population dynamic regime sets in. When the rearrangement probability is high, clusters break up until the average minimum arc distance settles on high values (Figure 1A). In these cases, the selfish operon model fails to maintain tight clustering in the long term. In particular, gene clusters do not evolve under the parameter values used by Lawrence and Roth [10].

**Figure 2:**
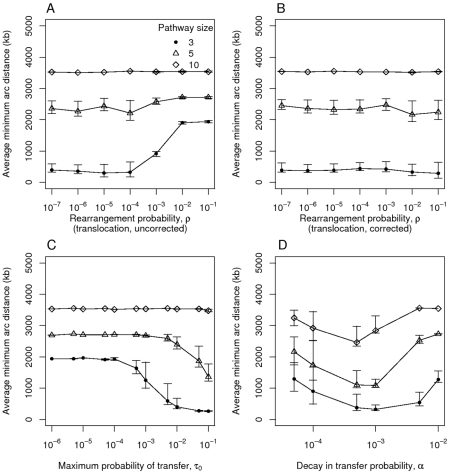
Gene clustering under the selfish operon model. The average minimum arc distance between genes at equilibrium as a function of various parameters: A) the probability of rearrangement 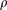 under the original uncorrected translocation process, B) rearrangement probability 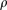 with the translocation process corrected so that the probability 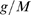 of choosing the genes in question is included, C) the maximum transfer probability 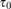; D) the parameter 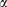, which describes the decay in the horizontal transfer rate over distance. Each point indicates the mean of 100 runs and error bars show the central 90% of simulations. Each simulation was run for 50,000 time steps. Unless indicated otherwise, there are three genes in the pathway and the parameter values are 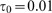, 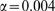, and 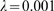.

To determine if there are conditions under which the selfish operon model *does* produce clustering, we re-examined this model by exploring its parameter space. Figure 2 reveals the effect of varying the parameters in this model on the average minimum arc distance. It shows that under the original model clustering is only produced when the rearrangement probability 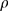 is low, the number of genes 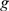 is small, and the maximum transfer probability 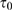 is sufficiently high. Under the corrected translocation process, the effective rearrangement probability is lowered by a factor 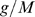 and the probability 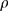 itself has no apparent effect on clustering. The decay in transfer probability 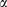 (see Equation 1) must take intermediate values of around 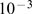 for clustering to evolve. If 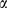 is too low, selection is too weak to promote clustering while if it is too high, the probability of transfer is depressed for most minimum arc distances, preventing selection from acting effectively.

**Figure 3:**
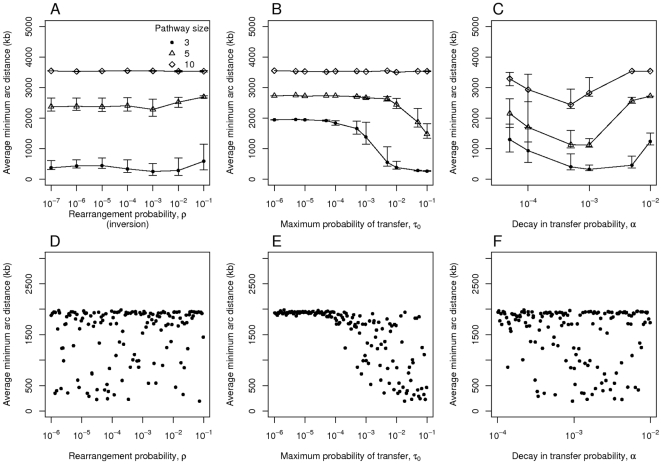
A sensitivity analysis for the selfish operon model with inversion rather than translocation. Each panel plots the average minimum arc distance between the genes. Simulations were run for 50,000 steps. In the top three panels (A–C) one parameter is varied at a time while keeping the others constant. Each point represents the mean of 100 simulations and error bars indicate the central 90% of simulations. The responses are shown for three different values of the number of genes, 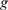. The plots show distances over the probability 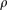 of rearrangement, which occurs here through inversion (panels A and D), the maximum probability of transfer 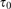 (B and E) and the decay in transfer probability over distance 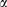 (C and F). The default parameter values for these simulations are 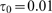, 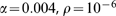 and 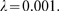 The bottom three panels (D–F) show the results of simulations for 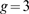 in which the parameters were set randomly according to latin hypercube sampling with 150 points and 40 simulations per point.

Very similar results are observed when translocation is replaced by inversion, as shown by varying one parameter at a time as well as by latin hypercube sampling analysis (Figure 3). The major difference is that a high probability of inversion does not prevent the evolution of clusters to the same extent as observed in the uncorrected translocation process of Figure 2A.

### Moran model

**Figure 4:**
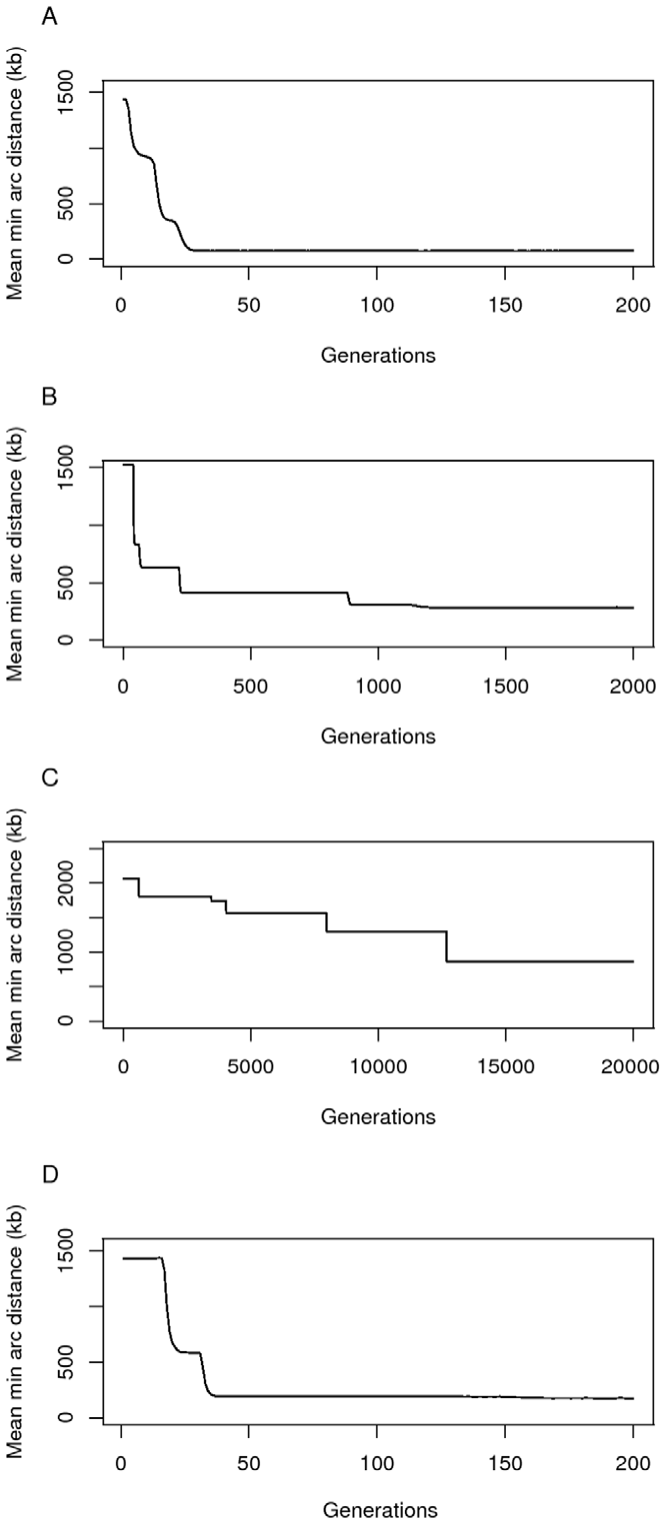
Gene clustering under a Moran model. The average minimum arc distance between genes over time for four sample runs of the simulation using rearrangement rates 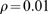 (A and D), 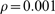 (B) and 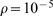 (C). The other parameters are 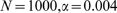 and 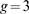 genes. In panel D) a run of the simulation is shown in which we model selection for distance using a sigmoidal instead of exponential function. In this case, fitness decreases markedly between distances of 5 and 20kb. The final distance after 200 generations was 176 kb. Observe that in panel B) it took more than 10 times as long for the genes to approach a clustered state (distance 284 kb) than in panel A) (distance 77 kb), and that in panel C) the genes are still far apart at around 850 kb after 20,000 generations.

We further explored the evolution of clustering using the Moran model with selection for gene clusters. By holding the population size constant this model also allows us to disentagle the effects of population dynamics from those of rearrangement and selection. Figure 4 shows simulation runs of the process for progressively lower values of 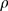: 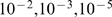. It was not computationally feasible to run the simulation under even lower, and more realistic, values. The general pattern emerging from these sample trajectories is that the minimum arc distance is reduced through a series of selective sweeps. The time taken until the appearance of a rearranged genome that reaches fixation is long and depends on the rearrangement probability 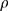 and the population size 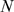. The reduction of minimum arc distance is a slow process made even slower by lowering 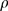. Using a steep sigmoidal function for selection instead of exponential decay (Figure 4D) gave qualitatively similar results.

### Rearrangement substitution model

**Figure 5:**
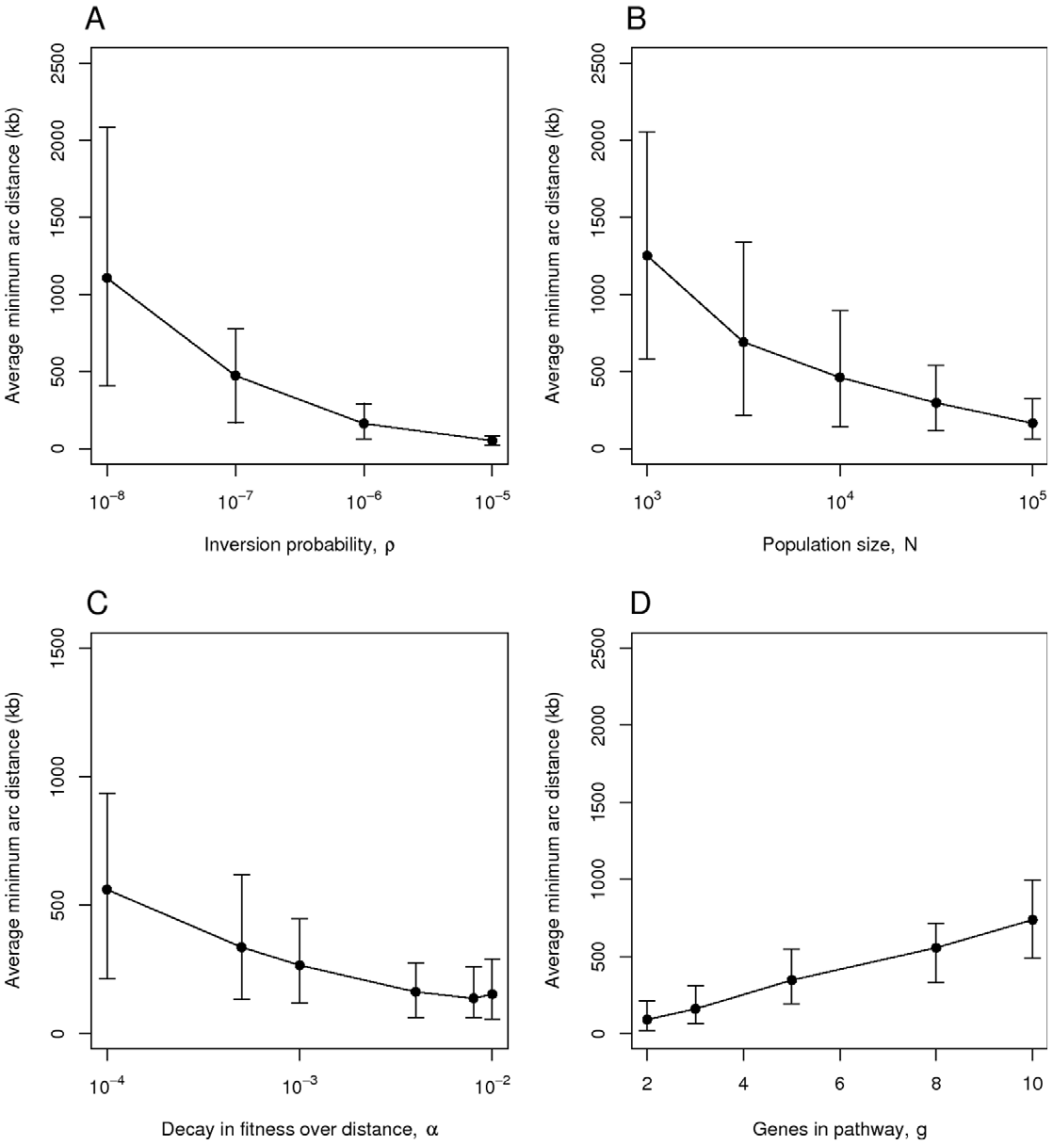
Rearrangement substitution model, varying one parameter at a time. The panels show the average minimum arc distance between the genes plotted over A) the inversion probability 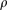, B) the population size 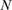, C) the decay in fitness over distance 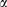 and D) the number of genes 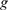 in the pathway in question. The default parameter values are 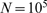, 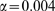, 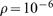 and 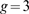. Simulations were run for 50,000 generations. Each point represents the mean from 100 simulations and the error bars indicate the central 90% of simulated values.

The rearrangement substitution model, which “compresses” time by tracking fixation events, is amenable to sensitivity analysis. Figure 5 demonstrates that a low rearrangement probability of 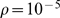 is able to produce clustering in 50,000 generations. Even lower probabilities lead to weak or no clustering because successful rearrangements that reduce the distance between genes are too rare. Increasing the population size 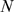 improves the efficiency of selection and leads to clustering. Similarly, increasing the decay in fitness 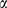 improves clustering. Gene clusters are also more readily formed for pathways with a smaller number of genes 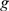.

**Figure 6:**
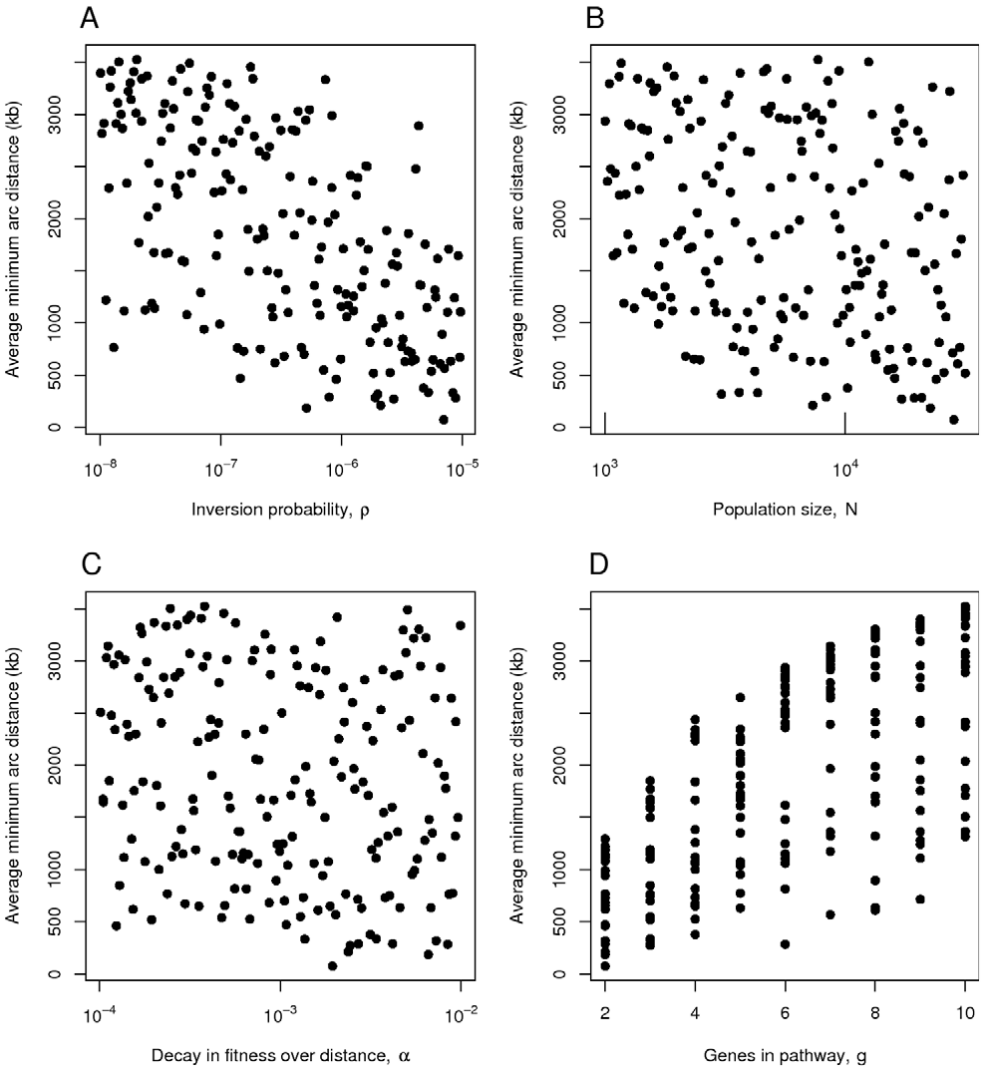
Rearrangement substitution model, varying the parameters of the model using latin hypercube sampling with 200 points. The panels show the average minimum arc distance between the genes plotted over A) the inversion probability 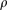, B) the population size 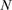, C) the decay in fitness over distance 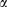 and D) the number of genes 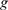 in the pathway in question. Simulations were run for 50,000 generations. Each point represents the mean from 100 simulations.

Similar results are produced when the parameter space is explored using latin hypercube sampling (Figure 6). Minimum arc distance decreases with 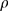 and 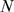 and increases with 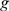. Distance also decreases with 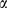, though this effect is subtle. For 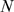 (panel B) and 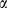 (panel C) the correlation with distance is statistically detectable using a non-parametric method (Kendall's tau), with 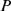-values of 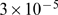 and 0.0148 respectively. The corresponding 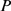-values for 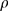 (panel A) and 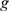 (panel D) were both less than 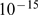. Note that each factor on its own does not explain much of the variation in distance.

## Discussion

This study presents new computational models showing that direct natural selection can lead to the formation of gene clusters under appropriate conditions. We have also re-examined an existing simulation model involving indirect selection – the selfish operon model. By exploring these models under many conditions, we have identified the regions in parameter space that produce gene clustering. In the following, we will discuss parameters as rates rather than probabilities per time step.

### 

#### Selfish operon model revisited and the role of rearrangement rates

The selfish operon model of Lawrence and Roth 1996 is able to produce gene clusters, but only when the right conditions hold. The overall transfer rate must be high, as reflected in the maximum rate of transfer, and the decay in transfer over distance must be in a suitable range. The rearrangement rate must be low. We note that with a slight correction – accounting for the probability that rearrangement affects the genes in question – these rates are indeed effectively low enough for clustering to evolve. When the selfish operon model is modified so that inversion is the mechanism of rearrangement, again gene clusters can evolve, and inversion rates must be low enough to prevent clusters from disintegrating too quickly once formed.

Rates of fixation of rearrangements are typically very low in nature. From comparisons of genomic data the number of rearrangements per genome per lineage varies across evolutionary lineages, but is usually on the same order of magnitude as or a little higher than the expected number of substitutions per site [37],[38]. Rocha (2006) found the substitution rate of rearrangements to be several orders of magnitude lower than the cellular per generation rate of 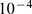 [39] because of selection against most new arrangements [40]. Overall these studies suggest the rearrangement rate may be on the order of 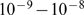 per year. The application of these figures to the model of Lawrence and Roth 1996 is not straightforward because the time unit is not set in that model. However, given the wide level of variation in gene content even within bacterial species [41] and the slow process of rearrangement [40], it is likely that the rearrangement rate is far lower than the rate of horizontal gene transfer. Our analysis of the selfish operon model suggests that gene clusters can evolve under such conditions.

#### A low number of genes in the pathway promotes clustering

In both the selfish operon model (Figures 2,3) and the rearrangement substitution model (Figures 5, 6), gene clusters evolved more readily when the number of genes in a pathway was low. It accords with intuition that less time is taken for a smaller number of genes to cluster. Yet large clusters exist in nature. A possible explanation is that clustering occurs in stages rather than all at once. For this scenario to work, subsets of genes already clustered must be held together while the remaining genes move close to the cluster. Biologically, what could make gene clusters an absorbing state? Clusters of genes are sometimes but not always transcribed and regulated together (found on operons). If such genes are separated, their function may be undermined or destroyed. Another possibility is that genes overlap on a chromosome [9]. If the region of overlap is essential to both genes, again selection would act to maintain the clustered arrangement of those genes. It is unclear, however, how widely applicable this mechanism is. Future modelling efforts could include the possibility that selection acts not only on the minimum arc distance but also on the particular arrangement of the genes. For instance, in a three-gene pathway, a genome in which two genes are close together may be favoured over one in which the three genes are spaced evenly over the same minimum arc distance.

#### The roles of selection and transfer bias in gene cluster evolution

As intuition dictates, the evolution of clusters also requires some kind of a bias favouring clusters, which can appear in the form of biased horizontal transfer (the selfish operon model) or natural selection for gene proximity (the Moran model with inversion, and the rearrangement substitution model). In each case the parameters must be appropriate to give natural selection or transfer bias their efficacy to produce gene clusters. In the selection model the population size needs to be high and the decay in fitness over distance must be sufficiently high. Under the conditions we studied, the evolution of gene clusters is expected to occur very slowly. However, billions of years have passed since the last universal common ancestor, providing ample time for gene clusters and operons to have evolved and to have been transferred between species.

We remark that in mathematical models of the level of detail presented here, including the selfish operon model of Lawrence and Roth [10], bias in horizontal transfer is indistinguishable from direct natural selection. The persistence model of bacterial gene clusters described by Fang et al. [12] represents another model of selection. There, deletions are more likely to destroy gene function when the genes are further apart on the chromosome. This is a form of negative selection acting against non-clustered essential genes. Both the selfish operon model and the persistence model involve a form of indirect selection, and we suggest that either direct or indirect selection, or both, are needed for clusters to form and be maintained. Current models do not and cannot separate these two phenomena. For example, although we specified the Moran model for a population of individual cells under direct selection for gene clustering, it is possible to interpret the same model as one tracking a population of species (as in the selfish operon model) in which selection is indirect, and in the form of horizontal transfer biased towards low minimum arc distances.

We did not attempt to discriminate between the alternative forms of selection or bias favouring clustering. Rather, we have shown that under appropriate conditions these models can lead to gene clustering. A systematic and formal comparison of alternative models is a remaining challenge, which may require a common mathematical framework for comparing the consequences of these alternatives. Although the selfish operon model has been questioned as the sole mechanism for the evolution of gene clustering, we believe it cannot yet be rejected as a contributor on either empirical or theoretical grounds.
